# SNIRF2BIDS: a GUI-based tool for converting functional near-infrared spectroscopy data to the Brain Imaging Data Structure in R

**DOI:** 10.1117/1.NPh.13.S3.S32603

**Published:** 2026-09-17

**Authors:** Raphaël Lorenz-de Laigue, Jennifer Svaldi, Philipp A. Schroeder

**Affiliations:** a University of Tübingen, Department of Psychology, Clinical Psychology & Psychotherapy, Tübingen, Germany; b German Center for Mental Health (DZPG), Tübingen, Germany

**Keywords:** BIDS, data sharing, R, conversion, open-source software

## Abstract

**Significance:**

Optical imaging with functional near-infrared spectroscopy (fNIRS) often produces complex datasets. The Brain Imaging Data Structure specification (BIDS) has evolved to harmonize fNIRS recordings and to facilitate data sharing, transparency, and reproducibility. However, the conversion of raw fNIRS recordings and associated metadata into BIDS format remains time-consuming and error-prone.

**Aim:**

We aim to present SNIRF2BIDS, an R package that streamlines the conversion of fNIRS data to the BIDS format through a graphical user interface.

**Approach:**

SNIRF2BIDS automates dataset restructuring, metadata organization, and file naming in accordance with the current BIDS specification. The SNIRF2BIDS-GUI requires R (≥4.0.0), including recent versions of shiny (≥1.13) and rlang (≥1.1.7). The backend for conversion configures Python and MNE-BIDS to be available for R via reticulate (≥1.45). After initial setup, the tool operates locally without requiring data transfer to online platforms.

**Results:**

We demonstrate the functionality of SNIRF2BIDS in a step-by-step tutorial, guiding users from raw fNIRS recordings to fully BIDS-compliant datasets.

**Conclusions:**

SNIRF2BIDS enables standardized organization of fNIRS datasets, thereby reducing manual work and minimizing the risk of human error. It fosters data sharing, thus supporting reproducible workflows and meta-scientific progress in the growing fNIRS research community.

## 1 Need for fNIRS Standardization Tools

Functional near-infrared spectroscopy (fNIRS) is a neuroimaging method that measures changes in the concentration of oxygenated (HbO) and deoxygenated (HbR) hemoglobin on the cortical surface by sending near-infrared light into cerebral tissue. Although modern fNIRS is still relatively young compared with established neuroimaging modalities such as electroencephalography (EEG) and functional magnetic resonance imaging (fMRI), there has been a steady increase in fNIRS publications since the beginning of the twentyfirst century.^1^ Given its portability and ease of application, neuroimaging with fNIRS extends the study of cognition and behavior, e.g., in cognitive control, physical movement, and social interactions to more naturalistic environments across different study populations (e.g., Refs. 2–4). In addition, fNIRS can easily be combined with EEG or systemic physiology measurements such as heartrate or blood pressure, enabling, for example, mechanistic research into cognitive neuroscience and brain–body interactions^2^ and applications in personalized digital healthcare.^5^

Recorded fNIRS data are often complex, comprising the raw signal and extensive metadata. The signal typically consists of two time-series corresponding to HbO and HbR for each source-detector channel (sometimes also total hemoglobin [HbT] as a contrast—or measurements from a third wavelength). Metadata is used to describe the experimental setup, which can range from single optodes to complex arrangements of hundreds of channels distributed over the cerebral cortex, each sampling hundreds of data points per second (e.g., Ref. 6). Although large-scale, multisession fNIRS studies are not yet common, they have the potential to generate increasingly complex hierarchical datasets, a scenario made feasible by the affordability and participant-friendliness of fNIRS.^2^

In line with ongoing efforts to harmonize neuroscience research data according to the FAIR principles—findability, accessibility, interoperability, and reusability,^7^ there is growing emphasis on sharing fNIRS datasets^8^ to increase the credibility and reproducibility of findings.^9^ Seamless access to such rich datasets would also facilitate meta-scientific approaches and enable secondary research use cases, such as machine learning and big data analyses across datasets^10^ or comparative studies of data processing pipelines.^11^ Despite the marked increase in fNIRS publications from 2000 to 2019,^1^ fNIRS data is, however, not yet commonly being shared. For example, the OpenNeuro database^12^ included 950 fMRI and 332 EEG datasets as of this writing, but only 17 fNIRS datasets.

To facilitate sharing and reproducibility, raw fNIRS data must be reformatted and organized according to established standards. It cannot be automatically shared or interpreted by other researchers or computational tools.^7^ Effective sharing of fNIRS data requires the availability of both the time-series data and comprehensive metadata, including sampling frequency and optode configuration. These files must be organized into a hierarchical folder structure to be unambiguously linked to the corresponding participant, session, and task.^13^

The Brain Imaging Data Structure specification for near-infrared spectroscopy data (NIRS-BIDS) provides a standardized framework for structuring fNIRS data and metadata^13^ and is supported by an expanding set of software tools that automate conversion,^14^ validation,^15^ and formatting, reducing manual effort and facilitating adoption in larger-scale studies. By enabling standardized and automated data organization, NIRS-BIDS not only improves reproducibility but also makes large, multisession, and multimodal fNIRS projects more feasible. Building on this need for systematic data structuring, the following section presents the NIRS-BIDS standard and existing software tools for converting and managing fNIRS datasets.

## 2 Presentation of (NIRS-)BIDS and Existing Software Tools

The BIDS has emerged as a community-driven standard to harmonize neuroimaging datasets. BIDS was originally developed for fMRI and EEG but has recently been extended to accommodate fNIRS.^13^ The NIRS-BIDS specification builds on the hardware- and software-agnostic shared near infrared spectroscopy format (SNIRF), which stores most relevant acquisition parameters and metadata.^16^ However, this information is not automatically structured according to BIDS templates.

**Fig. 1:**
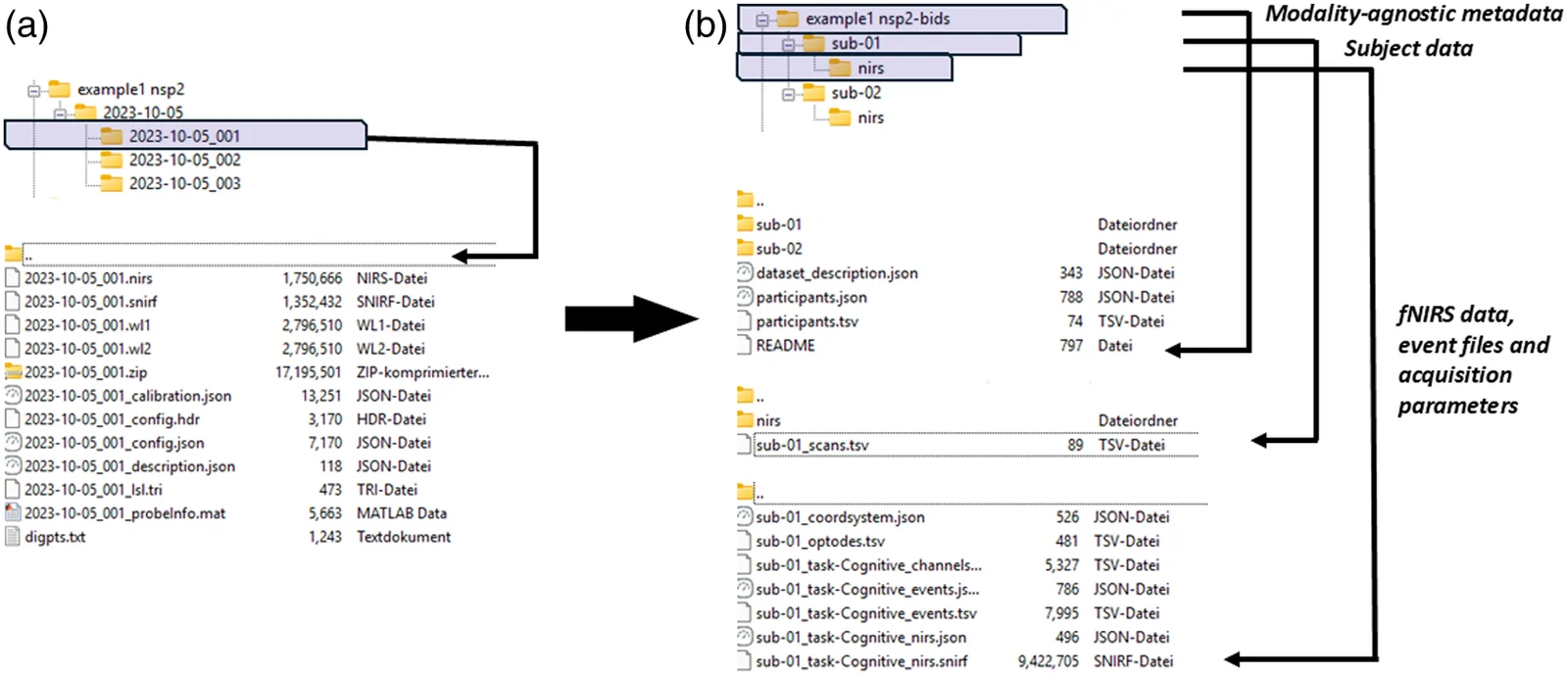
Illustration of an example conversion process from the original data structure to BIDS. (a) Raw fNIRS recordings in their original format as saved by the researcher. The folder structure (top) does not encode subject IDs or session information. A “*_description.json” file is available. The SNIRF recording file and metadata files are organized in a manufacturer-specific manner (bottom). (b) Converted fNIRS recordings in BIDS-compliant format. The folder structure (top) encodes subject IDs and session information (not shown in example) in a hierarchical structure. The subfolders at the level of dataset, subject, and data type are formatted in accordance with the BIDS standard and contain modality-agnostic, subject-specific, and measurement-specific metadata, respectively.

Saving fNIRS data into the NIRS-BIDS format requires metadata extraction, file restructuring, and systematic folder organization. The extracted metadata, such as sampling frequency or number of source and detector optodes, is stored separately from the SNIRF file in the BIDS-formatted data folder. Because BIDS encodes not only acquisition parameters but also study-level information on experimental design, task structure, and session organization, data from multiple participants and recording sessions must be organized into a nested folder structure^17^ (see Fig. 1 for an example).

Direct export to the BIDS format is currently uncommon in fNIRS recording software. BIDS rules can be applied via manual copying, pasting, and reformatting of the necessary information. However, BIDS conversion is a multistep process and research projects typically contain a large number of fNIRS recordings. Manual intervention can therefore quickly become time-consuming and hold potential for user errors.^18^ Alternatively, script-based workflows can be used to automate the conversion process via Python toolboxes such as Cedalion^19^ or MNE-BIDS.^20^ The MNE-BIDS conversion process is based on a single, powerful function (“mne_bids.write_raw_bids()), which is designed to reformat a single, raw fNIRS recording into BIDS-compatible format. The function does not only rename the file but also extracts relevant information into accompanying metadata files such as *_channels.tsv, *_nirs.json, or *_events.tsv. In Cedalion, the snirf2bids package incorporates different functions to read files from an input folder, specify the mapping to relevant information such as participant identifier or task name recording-by-recording in a *.csv file, generate appropriate file names and directory paths in the output folder, and create the relevant *.tsv and *.json files. Compared with MNE-BIDS, the workflow integrates a way to restructure whole datasets, through the mapping *.csv file. Moreover, the tool comes with a detailed step-by-step tutorial.^21^

Such workflows based on programming via command-line interface (CLI) not only allow flexibility and control, especially for advanced users, but also increase cognitive load for some users. When reformatting not only a single recording, but also a whole dataset, conversion scripts need to be conceptualized and iteratively optimized to match the structure of the raw fNIRS data. Alternatively, tools based on graphical user interfaces (GUIs) can allow for a more intuitive conversion process—a similar argument has been made on the conversion of MRI data.^18^ For fNIRS data, OpenfNIRS provides a GUI-based tool for the reformatting of recordings to adhere with BIDS standards on the BfNIRS cloud.^14^ The tool allows for automatic extraction of metadata from SNIRF files and assists the user in folder creation, renaming of files and providing missing metadata. However, files need to be converted one-by-one, which can get time-consuming for large datasets. Even more importantly, the use of a cloud-based tool requires data upload on an external server, which might not adhere with data protection policies across research institutions.

Overall, in the current ecosystem, existing software options for the conversion of fNIRS datasets to BIDS format have powerful features (see also Table 1 for an overview) but require either programming skills, especially for the conversion of large datasets, or data upload to external servers, which can limit accessibility and data privacy.

**Table 1:** List and features of open science tools for the conversion of fNIRS data (in the SNIRF file format) to BIDS-compliant datasets.

Tool	Cloud-based versus local	Backend language	Frontend user control	Instructions	Conversion of single recording	Conversion of multiple recordings	Link
BIDS fNIRS cloud	Cloud-based	Unknown	GUI	Online step-by-step tutorial	Default mode	Repeated conversion of single files	Ref. 22
MNE-BIDS	Local	Python	CLI	Online function documentation	One line of code	Requires scripting	Ref. 23
Cedalion	Local	Python	CLI	Online step-by-step tutorial	Requires scripting + manual editing of mapping file	Requires scripting + manual editing of mapping file	Ref. 24
SNIRF2BIDS	Local	R & Python	GUI	Local, integrated in the GUI + step-by-step tutorial	Possible via GUI	Possible via GUI; can require manual restructuring of input folder	Ref. 25

## 3 Our Contribution

We introduce SNIRF2BIDS, a GUI-based R package for converting fNIRS data into the BIDS format, which can be executed locally on the user’s machine. Overall, SNIRF2BIDS is a research tool which breaks the multistep process of standardizing fNIRS data to BIDS into small substeps. It produces standardized BIDS-compliant datasets, thereby facilitating reproducible workflows and enabling meta-scientific progress in the growing fNIRS research community. It may also fulfill an educational function in the dissemination of the BIDS standards.

SNIRF2BIDS expands the burgeoning ecosystem of software tools for standardization of fNIRS datasets by an approach targeted on users preferring a GUI interface and text-based instructions. The workflow is designed to convert whole datasets, rather than single recordings and also includes modules for the creation of modality-agnostic metadata such as the “dataset_description.json” and “Readme” files. Depending on the specific study design and user preference, our workflow might be less error-prone than manual interventions, respectively, less cognitively demanding than programmatic restructuring of datasets.

Our tool leverages MNE-BIDS as a backend component while providing an additional abstraction layer through a Shiny-based GUI interface in R, thereby lowering the barrier to BIDS adoption for researchers already working within that programming environment. Similar to Python, R is a widely used, open-source language for statistical analysis in psychology and neuroscience.^26^^,^^27^ Research labs may have established preferences for one environment over the other,^28^ and, to our knowledge, no R-based solution for the BIDS standardization of fNIRS datasets has been available to date.

In contrast to cloud-based solutions, the R-based approach does not require streaming raw data over the internet, which may better align with institutional data protection policies and individual preferences. Once it has been configured, SNIRF2BIDS offers a practical and attractive solution for research laboratories who favor a fully on-site workflow.

To promote accessibility and adoption, this paper provides a step-by-step tutorial that guides users from raw fNIRS recordings to fully BIDS-compliant datasets.

## 4 Installation and Setup

SNIRF2BIDS runs as a standalone R package and must be installed directly from its GitHub repository:^25^

pak::pkg_install("raphael-lorenzdelaigue/snirf2bids”)

Alternatively, a distributable package file is can be downloaded from the Open Science Framework (^29^) (e.g., to drive “E:/”) and installed locally:

install.packages("E:/SNIRF2BIDS_1.0.tar.gz", repos = NULL, type = "source")

It is recommended that users update all out-of-date packages on their machines prior to installation, as out-of-date packages led to compatibility issues in our test setups. A convenient call to do so is

update.packages(ask = FALSE)

Internally, SNIRF2BIDS also uses functions from the Python-based MNE-BIDS package.^20^ Miniconda,^30^ called within the reticulate package,^31^ is used to setup a Python environment (Version 3.10) during the package installation process. After installation, the converter can be started with a simple command

library(SNIRF2BIDS)

start_snirf2bids()

If script-based interaction is preferred as an alternative approach to the GUI, we do provide a manual installation guide for R access to MNE-BIDS functions in the Supplementary Materials (Code S1).

## 5 Main GUI and General Workflow

**Fig. 2:**
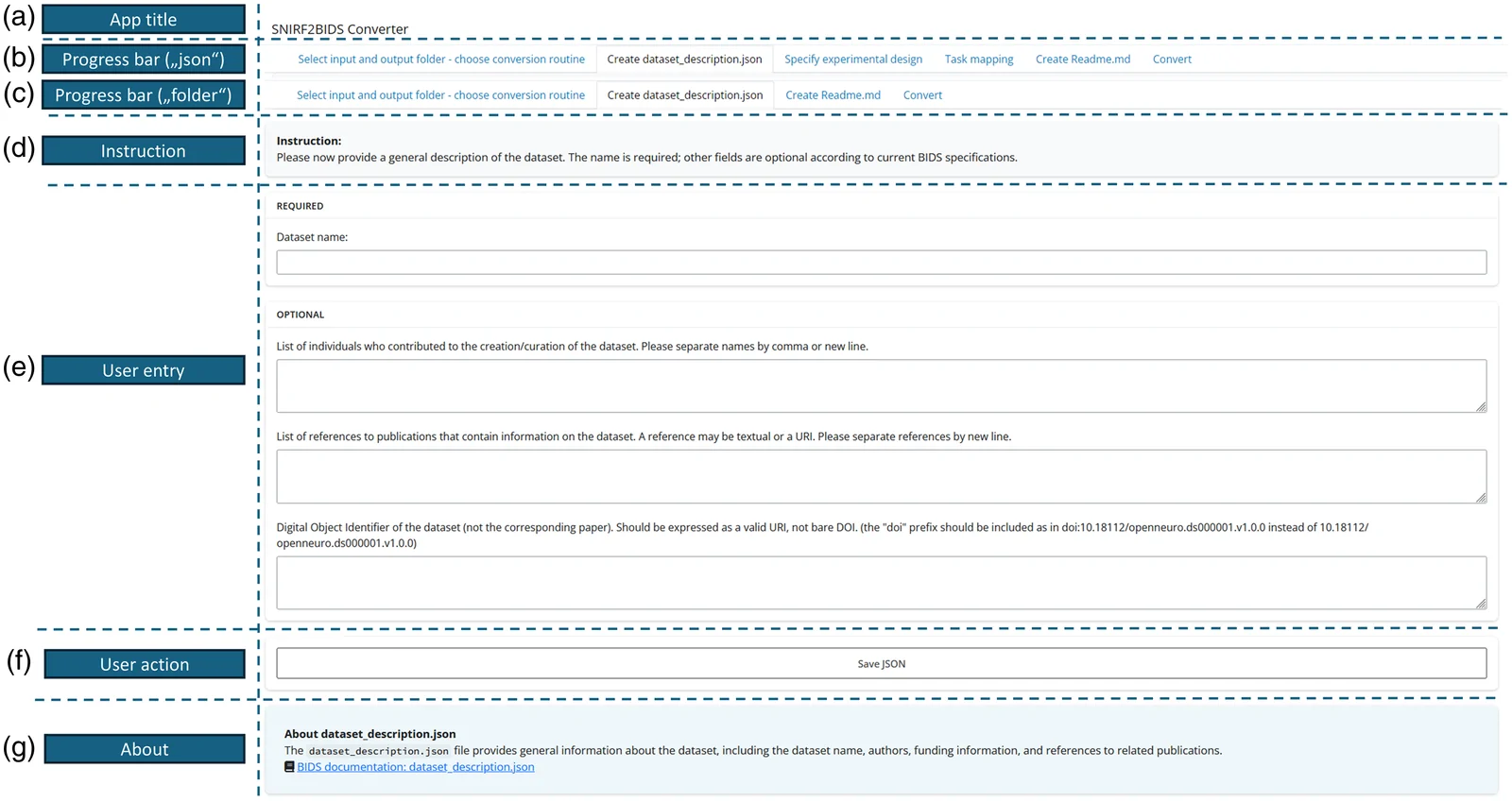
Overview of the GUI components. (a) App title. (b) Progress bar indicating the current conversion step, as presented in the tutorial (“json” routine). (c) Progress bar indicating the current conversion step, as presented in the tutorial (“folder” routine). (d) Brief instruction guiding the user through the conversion process. (e) Fields for entering user data. (f) Action button to complete the current conversion step. (g) “About” section containing background information on the current conversion steps. Links to relevant parts of the BIDS documentation.

Through the GUI (Fig. 2), users can monitor which step of the conversion process is currently active. The app is structured around a sequence of conversion steps building upon one another. We describe the required steps in detail below. For an overview, first, as a general setup (step 1), the user selects the input folder (containing fNIRS recordings to be converted) and output folder (where the BIDS-formatted files are stored). The user then chooses whether the subject ID, session number, and task name should be determined from the input folder structure—corresponding to the internal “folder” routine—or from the accompanying “*_description.json” file provided alongside the SNIRF file for certain manufacturers, corresponding to the internal “json” routine. Furthermore, a general description of the dataset is provided (step 2). If the “json” routine is chosen, the user can specify their experimental design and task mapping; the nested folder structure and file-naming scheme are hereby specified (steps 3 and 4). If the “folder” routine is chosen, these steps are skipped and will not be shown in the GUI. The user can then create the “Readme.MD” file, which is saved at the root directory of the project (step 5). Finally, using MNE-BIDS, fNIRS-specific metadata is extracted from the SNIRF files into the BIDS-compliant metadata files and copied, together with the SNIRF files themselves into the hierarchical data structure (step 6).

## 6 BIDS Naming Conventions

Beyond the general idea of BIDS, its detailed specifications include a substantial amount of (technical) definitions, terminology, and conventions that can be overwhelming to consider. For example, according to the BIDS specifications, identifiers for subject, session, and task should be composed exclusively of alphanumeric characters—that is,

– lower case or upper case letters from the latin alphabet (a-z and A-Z)
– numbers from 0 to 9

In general, special characters should not be part of identifiers. If necessary, it is possible to use “+” but all other special characters, especially hyphens (“-“) and underscores (“_”) should be avoided, as they might interfere with the standardized filename structure. When using numbers, zero-padding is recommended to ensure proper alphabetical sorting. That is, identifiers should preferably be written as “01,” rather than “1.” It is also important to avoid identifiers that only differ in in letter case within the same dataset. For example, a dataset cannot contain both “sub-s1” and “sub-S1”.”

## 7 General Setup

### 7.1 Preliminary Step: Assessing the Structure of the Input Folder

At the implementation level, SNIRF2BIDS needs to determine the subject ID, session number, and task name for each SNIRF file from the input folder. According to the official SNIRF specification, subject ID is a minimal required field in the metaDataTags entry and should therefore be readable within the SNIRF file itself. However, in some of our test recordings, this field was left empty by the manufacturer. Other fields, such as session number and task name, were also not provided in our test data within the SNIRF file itself.

Therefore, we designed SNIRF2BIDS to infer these values in one of two ways—either from the input folder structure or from the accompanying “*_description.json” file, when available. The “*_description.json” file contains the necessary metadata but is not being generated across manufacturers of NIRS devices. If no “*_description.json” file is available, the user needs to manually restructure the input folder before the conversion process into a nested folder structure.

### 7.2 Creating the Input Folder Structure (without Metadata File)

If using the “folder” routine, a separate subfolder needs to be created to serve as the input folder for SNIRF2BIDS:

– for each subject, named after her/his identifier (e.g., “AB123”)
– within each subject subfolder, a new, labeled subfolder for each measurement session (e.g., “AB123/01,” “AB123/02” etc.),
– within each measurement session, a new subfolder for each NIRS task (e.g., “AB123/01/task1,” “AB123/02/task2” etc.)

Importantly, all identifiers for subjects, session, and tasks should be composed exclusively of alphanumeric characters or, if necessary, the special character “+” (see also the section on “BIDS naming conventions).

In summary, the raw data need to be restructured into a hierarchical folder structure following the hierarchy subject-id > session-number > task-name. During the internal conversion routine, SNIRF2BIDS will then automatically read the relevant information from the subfolder names.

### 7.3 Step 1: Select Input and Output Folders, Describe Structure of Input Folder

As a preliminary step, the user must define two directories: (1) the input folder containing raw measurement data to be converted to BIDS and (2) the output folder, which will harbor the formatted folders and files. In the presented version of SNIRF2BIDS, both folders need to be located on a local hard drive as network drives may not be detected by the shinyFiles viewer when running locally.^32^ In the subsequent conversion process, SNIRF2BIDS will scan all SNIRF files found in the input folder. It is also important that the recordings are provided in the SNIRF format. The SNIRF file format has become the standard for fNIRS recordings and setups; other tools can be used to convert fNIRS-data recorded in other formats to SNIRF (e.g., Homer3, MNE, pysnirf, FieldTrip).

In the first step, the user also specifies whether SNIRF2BIDS should scan for “*_description.json” files alongside the SNIRF recordings—corresponding to the internal “json” routine—or extract the experiment information from the folder structure in the input folder, which corresponds to the internal “folders” routine. That is—at the implementation level, our tool automatically infers the subject ID, session number, and task name for each SNIRF file from either the “*_description.json” or from the structure of the input folder.

### 7.4 Step 2: Dataset Description

As a modality-agnostic requirement for compliance with BIDS, we include an option to create the “dataset_description.json” file. The only required entry that needs to be provided here by the user is the name of the dataset. Our tool also includes the option to specify authors, associated publications, and dataset DOI, which, as we believe, can further increase transparency in the sharing process and usability of datasets. These fields are only optional according to BIDS, so they can be left blank without impairing the further conversion process.

## 8 Define the Hierarchical Folder Structure and File-Naming Scheme

### 8.1 Step 3: Define Experimental Setup

If the “json” routine was chosen, the user now specifies the study design, which is automatically inferred from the input folder structure in the “folders” routine. First, the user indicates whether the study involves one or multiple sessions. A session is generally defined as an appointment during which data—fNIRS or otherwise—has been recorded for a specific participant. The user may define two separate sessions even if they took place on the same day, if this aligns with the specific study design (for example, if a recording takes place in the morning, the participant then sleeps and then another recording takes place in the afternoon). The user then declares which sessions contain fNIRS data, and how they wish to name the according tasks. Task names must be specified for each session where fNIRS measurements were taken. If multiple fNIRS recordings are part of the same session, the different task names must be separated by a comma.

Overall, our approach accommodates a wide range of experimental designs—from single-session experiments to longitudinal studies—with a freely specifiable number of fNIRS tasks. Internally, this step defines the nested folder structure and generation of BIDS-compliant filenames for the current project. During conversion, a corresponding subfolder is created in the subject-specific subdirectory of the output folder for each experimental session. Task names and session numbers are incorporated into the filenames of the converted recordings. Under the hood, the user also generates a temporary comma-separated value file (*.csv) containing the entered information by confirming the experimental design. The file is named “dataset-name_tasks.csv,” where the dataset name is read directly from the information provided in step 2 and is necessary for the creation of a new task mapping (step 4).

### 8.2 Step 4: Task Mapping

In the following window, the user specifies how the recordings corresponding to each fNIRS task defined in step 3 have been annotated in the “*_description.json” files found in the input folder containing the original source data. This interface uses the datamods GUI framework^33^ within Shiny for *.csv editing. The GUI displays the session numbers and task names defined in the previous step. A new column is offered to enter the task name to scan for in the folder containing the original recordings. The goal is to correctly identify, for each fNIRS recording and subject, the source file that will be converted. Under the hood, the resulting mapping of task names in the source files to BIDS-compliant session number and task name is saved as a temporary *.csv file named “dataset-name_tasks_mapped.csv.”

## 9 Readme File

### 9.1 Step 5: Readme.MD

We also provide a GUI for generating a Readme.MD file. The goal of the Readme file is to capture information that is not provided by other files in the BIDS dataset—or that is important to be repeated to external users who are analyzing the data. The content of the Readme.MD spans a lot of the information that would be expected in the Methods section of an experimental paper and includes, amongst others, an overview of tasks in the dataset, a description of relevant variables (dependent, independent, and control), relevant recruitment procedures, inclusion and exclusion criteria, and notes on missing data. We include the freely available BIDS template to guide SNIRF2BIDS users into providing all relevant information about the dataset.

## 10 Conversion

### 10.1 Step 6: Initiate Conversion Process

During the actual conversion process, SNIRF2BIDS extracts the relevant metadata (such as sampling frequency, optode count, and timing of task events) from the SNIRF files into the BIDS-compliant metadata files, such as the sidecar JavaScript Object Notation file (JSON) (*_nirs.json) or channels description (*_channels.tsv), using MNE-BIDS.^20^ The recordings are then copied, alongside the reformatted metadata, into the nested folder structure. Recordings are detected for each combination of participant, session, and task based on the user-defined file naming system from the input folder. For example, if two fNIRS recordings (*task1* and *task2*) took place during session 2 of a study with N=100 participants, SNIRF2BIDS will try to find the corresponding 200 SNIRF recordings in the input folder, extract the metadata, and rename the files according to the BIDS nomenclature—taking the provided task name and session number into account—and then automatically copy all converted files into the nested output folder structure.

Subject IDs and session numbers are being inferred either from the input subfolder structure or from information entered in the detected “*_description.json” files, combined with the task mapping provided by the user in steps 3 and 4. In compliance with BIDS standards, a new subdirectory is created for each study participant in the output folder (“sub-xxx”). Within subject-level subfolders, a subfolder is created for each recording session (“ses-xxx”).

## 11 Handling Files that Could Not Be Converted

It is possible that a dataset contains SNIRF files that are missing required fields or have an invalid file structure. Such cases may be detected directly by the “mne_bids.write_raw_bids()” function or any other function involved in the conversion process. Error messages appearing during conversion are being displayed in a red notification box at the bottom-right corner of the GUI and collected in a logfile (“log.txt”) placed at the top-level directory of the output folder. The GUI also provides feedback on whether the conversion loop ran until the end (“Conversion complete!”) or not (“Conversion failed”).

For the “json” routine, recording files with task names not defined in the task mapping overview are automatically copied to a separate subfolder named “no_mapping.” These files are tagged with the session number “999.” At the end of the conversion process, the user should review this folder to determine why some task names were not detected, which is often due to typos, and then move or rename these files as needed. Invalid IDs can be identified by reviewing the provided “unmapped_recordings.csv” file or subject-level subdirectories in both the successfully converted files and the “no_mapping” folder.

## 12 BIDS Validation

Next, compliance with the BIDS specifications can be validated using an external BIDS validator.^15^ This tool checks whether a dataset is compliant with the BIDS standard and provides technical feedback. It can be accessed via GUI in a web-based interface; in that case, the anonymized dataset needs to be uploaded on the tool website. Alternatively, for local use, CLI-based versions are available in Python or JavaScript.

After running the check, a BIDS compliant dataset should not return any “Error” feedback. Regarding “Warning” feedback, the validator may provide detailed feedback about missing metadata entries. In our end-to-end example, we include a section explaining the different warnings that appear when validating our test dataset. BIDS-compliant datasets without “errors” or “warnings” will yield “success” feedback. After validation, the data are ready to be shared in an open repository.

## 13 End-to End Example

In the following section, we present the necessary steps to convert a test dataset of N=3 individuals who completed a baseline session (a localizer task in session 1 and an experimental task) as well as two neurofeedback sessions. Data were recorded using a NIRSPort2 (NIRx Medizintechnik GmbH, Berlin, Germany), combined with the recording software Aurora. With this manufacturer, a “*_description.json” file is automatically created alongside each measurement, which contains the necessary metadata for running the SNIRF2BIDS “json” routine.

### 13.1 Step 1

**Fig. 3:**
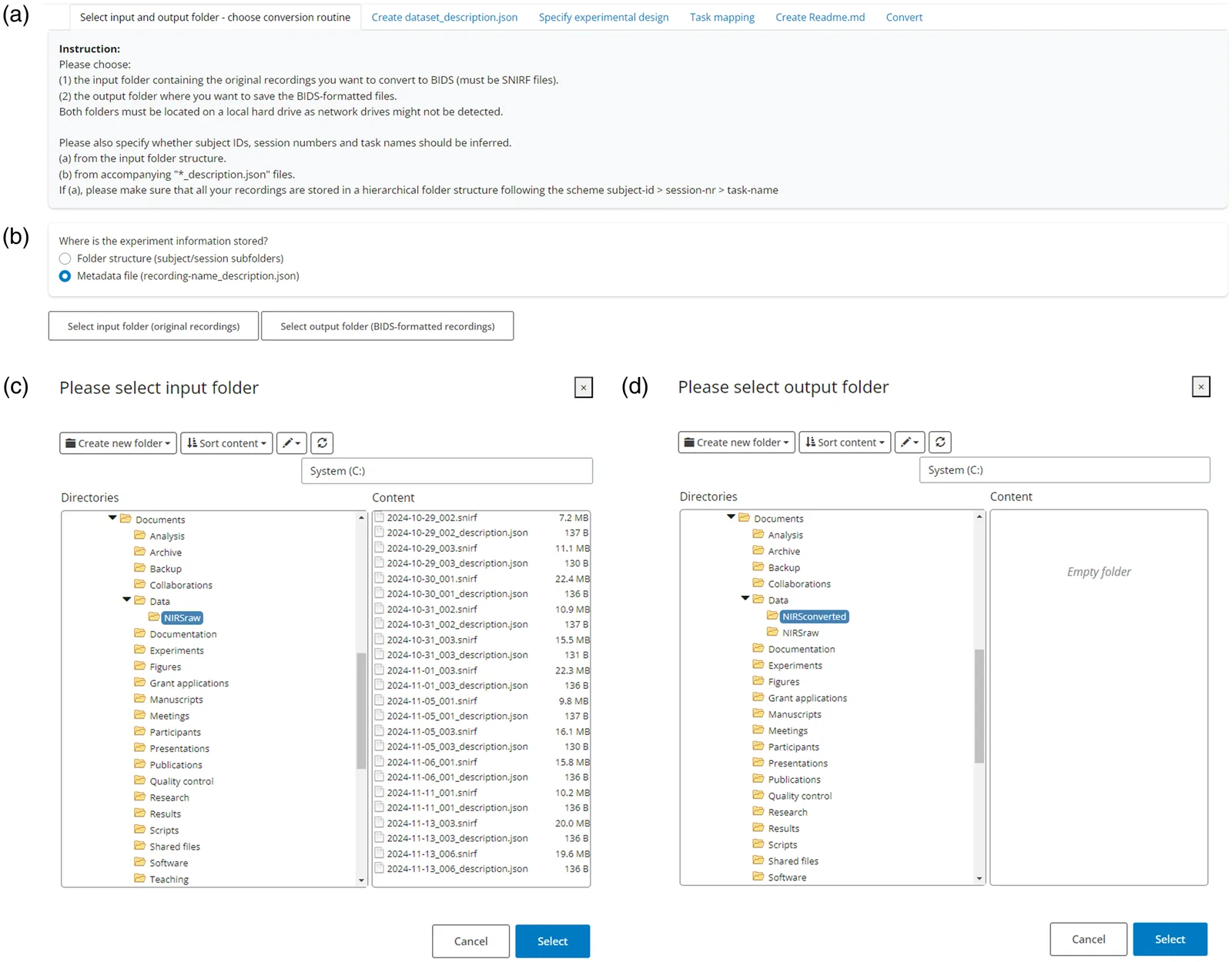
Selection of the output folder and conversion routine. (a) Instruction text displayed at the top of the graphical user interface (GUI). (b) Selection of the conversion routine, which also determines whether steps 3 and 4 are shown in the progress bar. (c) GUI dialog for selecting the input folder. (d) GUI dialog for selecting the output folder.

First, we indicate that the experiment information is stored in the metadata file. As a next step, we select the input folder containing the raw recordings to be converted. All local directories are listed in the left-hand pane of the file viewer (“Directories”). The user can navigate through the folder structure by clicking the black arrows and preview the content of the respective folder on the right-hand side of the pane (“Content”). Once a fitting folder has been selected, the user confirms the choice by clicking on “Select” at the bottom-right of the file viewer. The choice is confirmed by a notification on the bottom-right corner of the main SNIRF2BIDS GUI (e.g., “Source folder set to C:/Documents/my_input”). The identical workflow is performed a second time to choose the output folder. The user may also choose to create a new, custom-named output folder directly in the file viewer GUI. This choice is also confirmed in the main SNIRF2BIDS GUI by a notification (e.g., “Target folder set to C:/Documents/my_output”). This step is visualized in Fig. 3.

### 13.2 Step 2

**Fig. 4:**
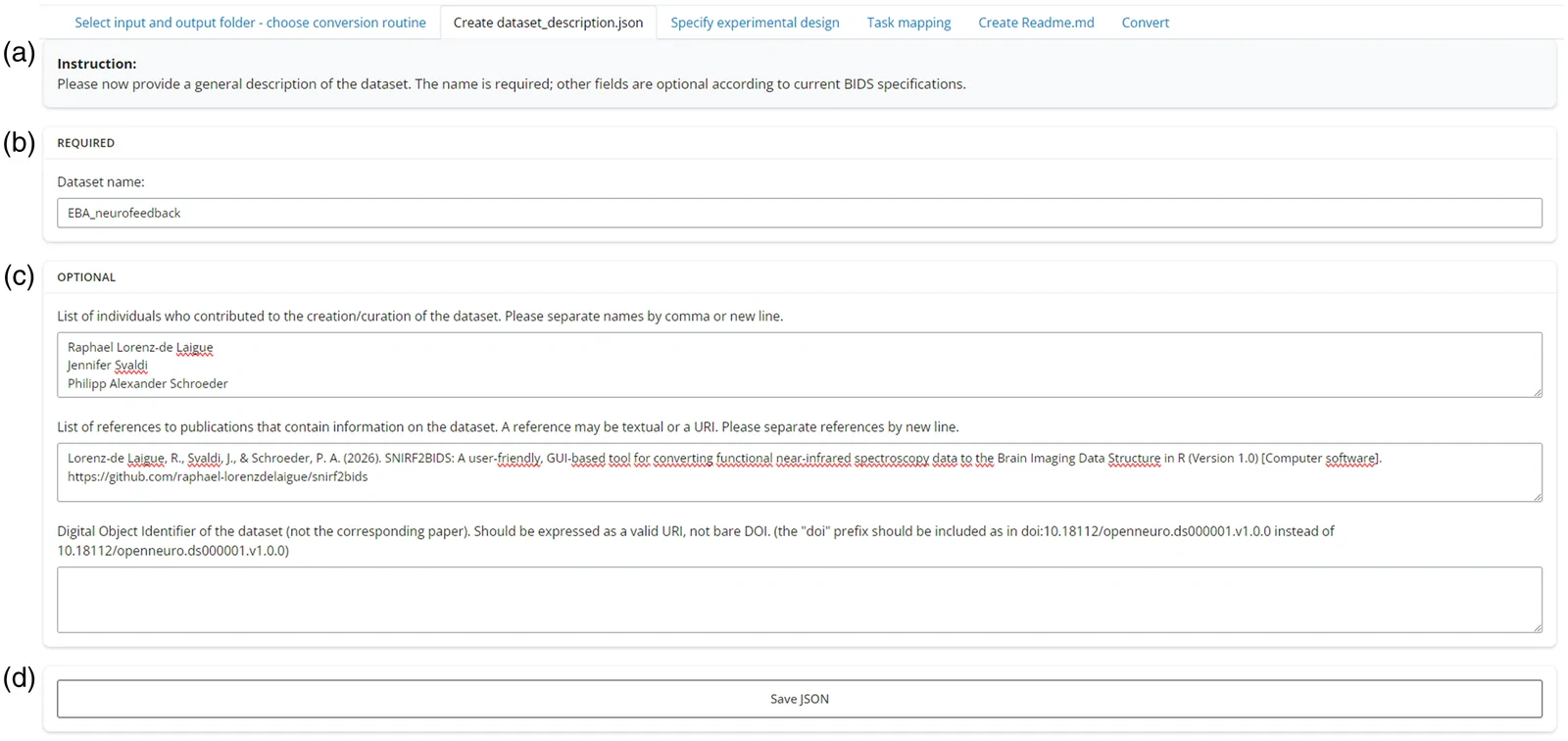
Input fields for creating the dataset_description.json file. (a) Instruction indicating that the dataset name is the only required field according to BIDS specification that needs to be entered by the user, whereas all other fields are optional. (b) Input field for the dataset name. (c) Input fields for optional metadata entries. (d) Button to confirm and save the JSON file to the output folder.

As depicted in Fig. 4, the user now must provide a name for the dataset. We choose a name that is not only short but also verbose enough that can be understood quickly (“EBA_neurofeedback”). In addition, some general information about the study can be provided—individuals who contributed to the creation of the dataset, references to publications containing information on the dataset and a digital object identifier. In our example, we provide the list of authors and publications corresponding to the SNIRF2BIDS repository, as our example data are not part of any other publication. Specific instructions are included in the GUI, guiding the user to correct the formatting of these data. When completed, the provided information is saved as the “dataset_description.json” file.

### 13.3 Step 3

**Fig. 5:**
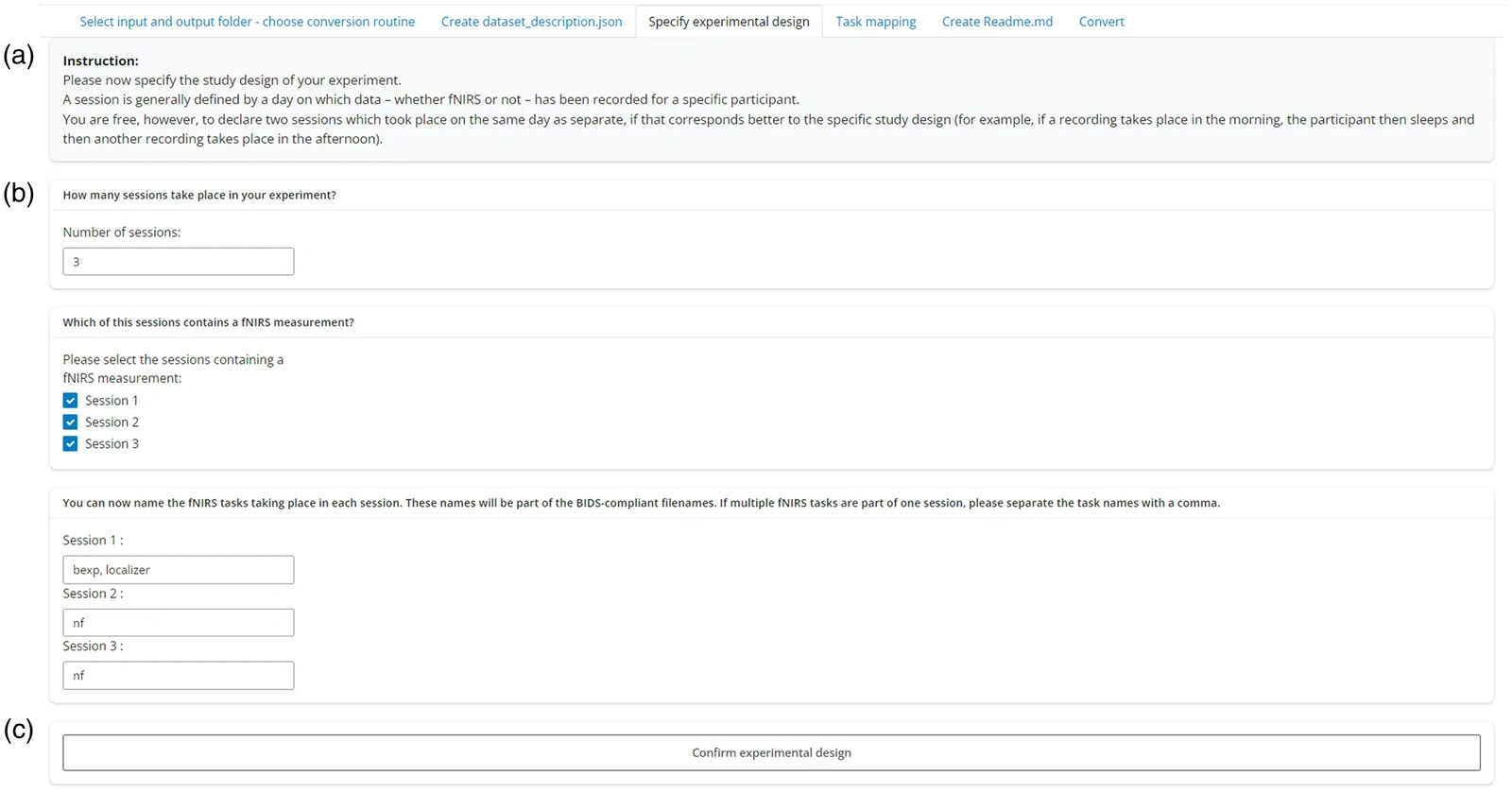
Specification of the experimental design (required only when using the “json routine”). (a) Instruction for defining the session structure. (b) Input fields for defining the total number of experimental sessions, indicating which sessions include fNIRS measurements, and assigning names to the corresponding tasks. (c) Button to confirm the experimental design.

The user now specifies the experimental design of the present study. As previously mentioned in the description of the example dataset, we indicate a total of three sessions, each one of them containing at least one fNIRS measurement. For each session, fNIRS recordings can be assigned labels that are automatically incorporated into BIDS-compliant filenames during the conversion routine. To improve readability, we compressed long words to acronyms (session 1: “bexp” for “body exposure” and “localizer,” sessions 2 and 3: “nf” for “neurofeedback). Depending on the user’s preferences, full names are an absolutely viable alternative. In any case, it is important that the labels are composed exclusively of letters and numbers. Special characters, especially hyphens (“-“) and underscores (“_”) should be avoided. Only “+” can be used as a special character, if necessary (for more detail, please refer to the section on “BIDS naming conventions). Figure 5 illustrates the experimental design specification.

### 13.4 Step 4

**Fig. 6:**
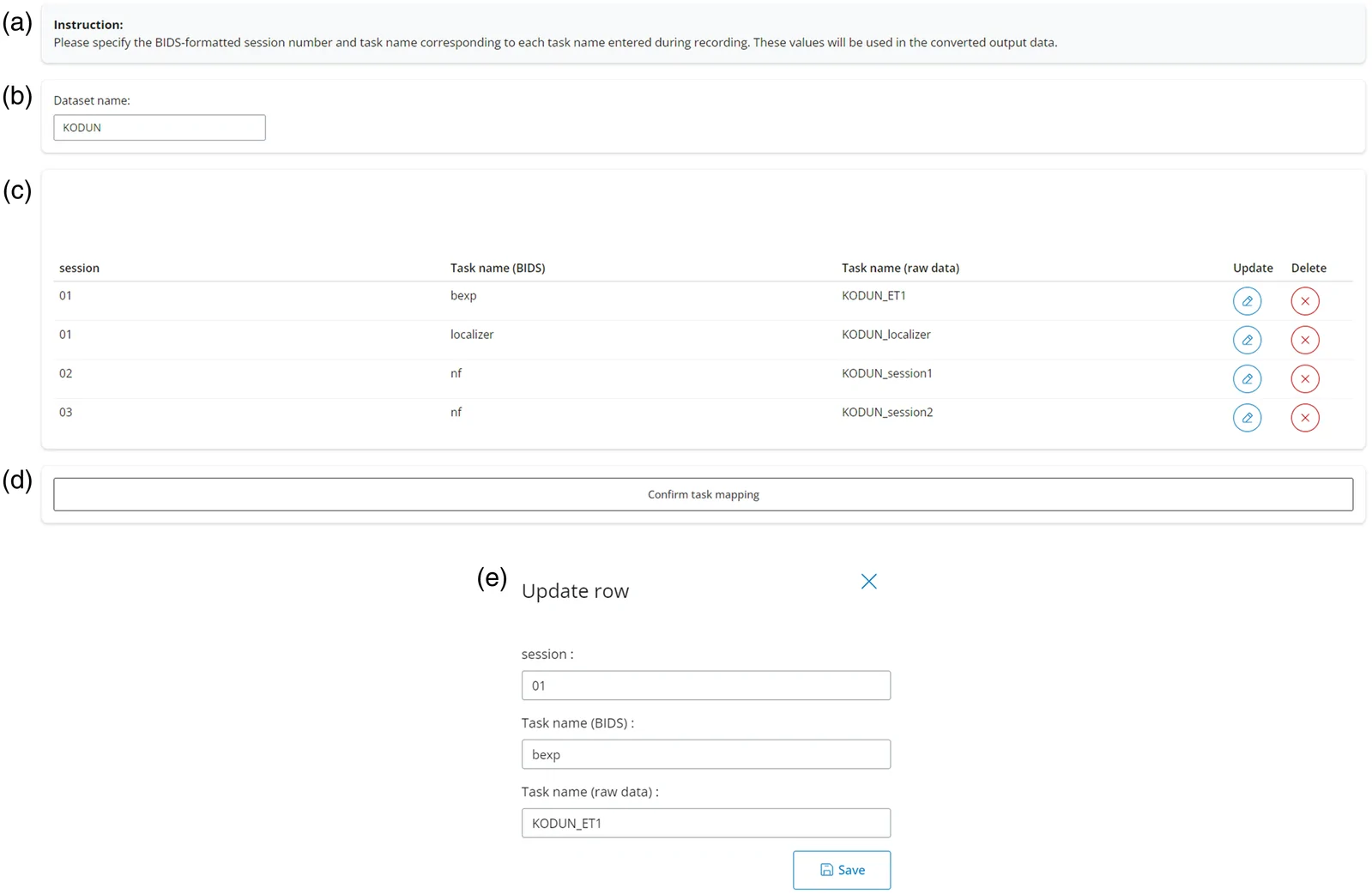
Mapping the BIDS-formatted session number and task name to the task name entered during recording. (a) Instruction text. (b) Input field for the dataset name, which loads session numbers and task names defined in the previous step. (c) Overview of mapping information, automatically displaying the current entries. Entries can be edited or deleted using the controls in the right-hand panel. (d) Button to confirm the task mapping. (e) Dialog window for editing an existing entry.

Now that the user has chosen labels for all fNIRS recordings in the study design for the BIDS-formatted filenames, the corresponding task name provided in the raw input data needs to be specified (Fig. 6). For example, during recording, we labeled the first neurofeedback session of a participant “KODUN_session1,” the second session was named “KODUN_session2.” These labels are saved in the *_description.json file during the recording and will be used by SNIRF2BIDS to locate the correct input files to be converted. In the GUI, entries can be updated by clicking on the pencil-shaped button. A new small text entry field then appears (“Update Row”), where the task name from the original recording can be provided (“task name (raw data)”). Once finished, the user can confirm the task mapping and proceed with the next step.

### 13.5 Step 5

**Fig. 7:**
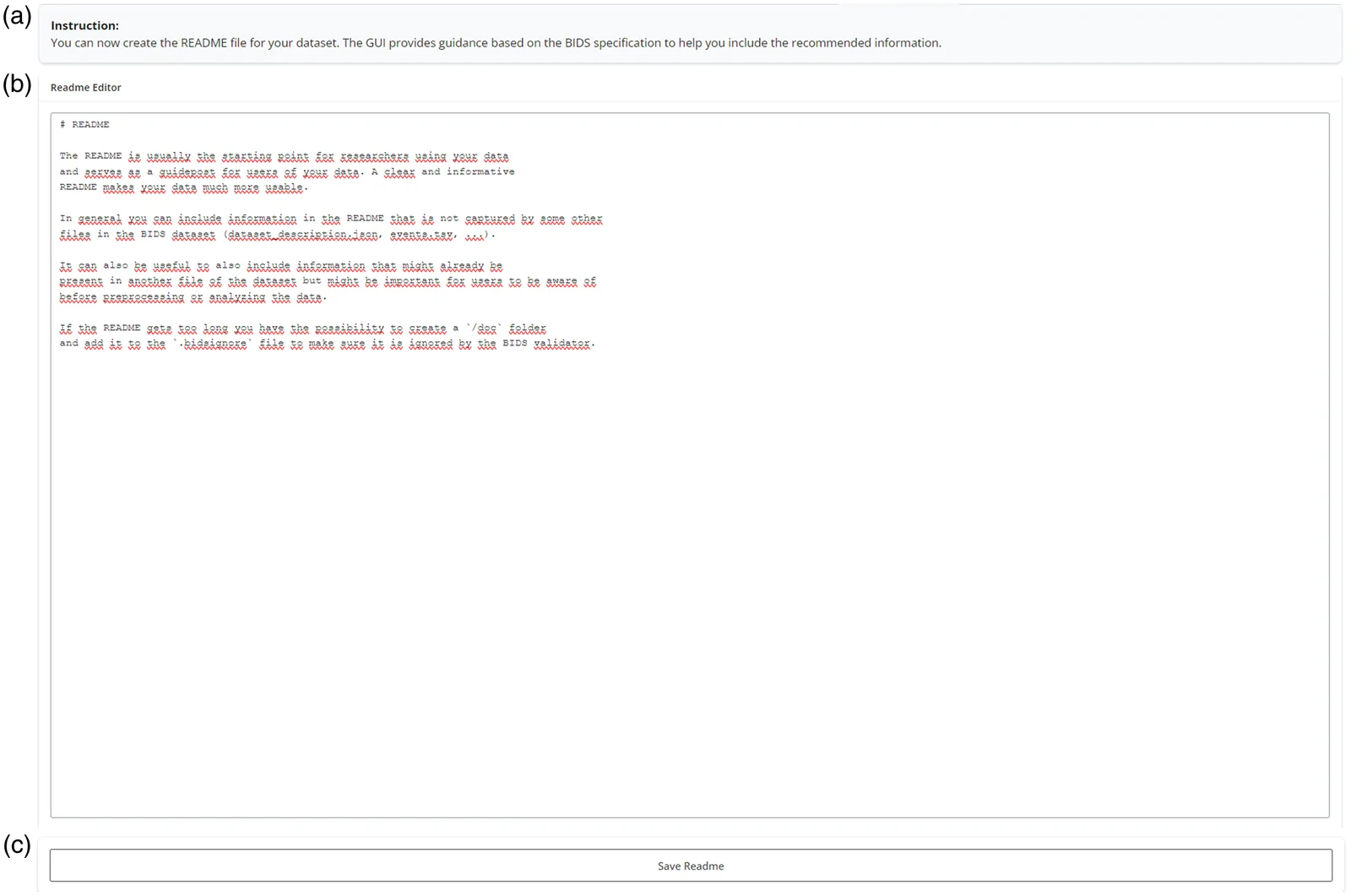
GUI for creating the “Readme.MD” file. (a) Instruction text. (b) Text input field pre-filled with BIDS-compliant README.md content (shortened in the figure). (c) Button to generate the file.

The user can now create the Readme.MD file according to the text-based description provided directly in the GUI. The description walks the user through the file, starting from general comments and then following with different sections of the document. The document should start with information about data access, followed by a general overview of the study design (e.g., brief summary of main tasks, description dependent and independent variables) and finally a more detailed description of study methods (e.g., subjects, measurement apparatus, detailed description of tasks). When finished with editing the file, the Readme can be saved via the GUI ("Save Readme“). The GUI is depicted in Fig. 7.

### 13.6 Step 6

**Fig. 8:**
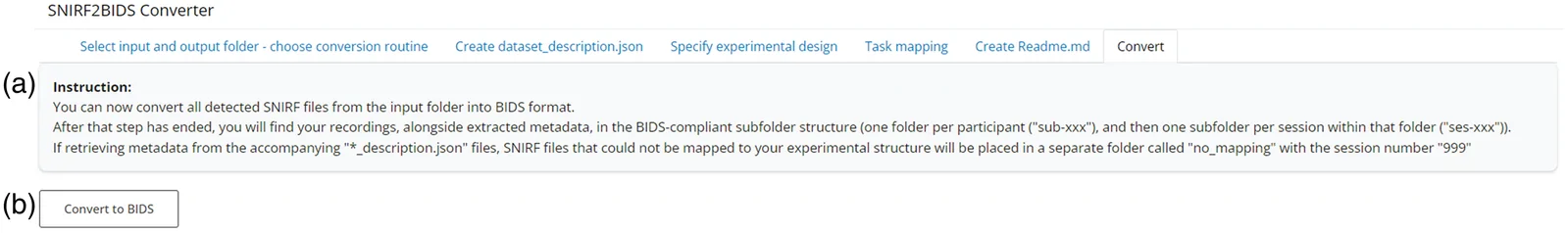
Starting the conversion process. (a) Instruction, describing the structure of the output folder and mentioning the “no_mapping” folder. (b) Button for starting conversion.

The user can now start the conversion process by simply clicking “Convert to BIDS” (see also Fig. 8). While the conversion is in process, a small dialog box is open on the bottom right hand of the app, indicating that the conversion is running. At the end of the conversion, the user is notified that conversion has been completed.

### 13.7 Handling Files that Could Not Be Converted

**Fig. 9:**
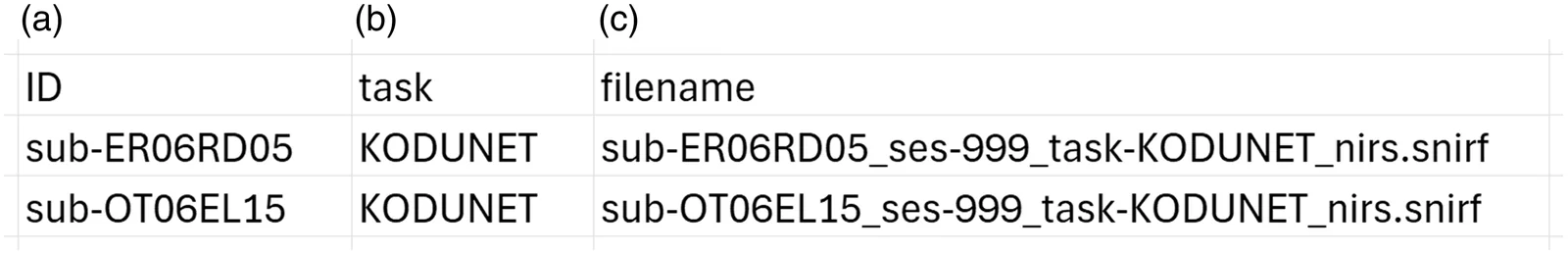
Overview of SNIRF files that could not be assigned to an experimental session or task using the “json routine” within “unmapped_recordings.csv.” (a) Subject identifier. (b) Task name entered during recording. (c) SNIRF file name as stored in the “no_mapping” folder.

After conversion, the user can check whether a logfile placed at the top-level directory in the output folder (“log.txt”) contains potential error messages having appeared during the conversion process. In the case of using the “json” routine, the user also needs to check manually whether any files are found in a folder named “no_mapping” in the output folder. For an overview, the folder contains a file called “unmapped_recordings.csv,” which lists all files that could not be assigned to a specific task or session (see Fig. 9).

**Fig. 10:**
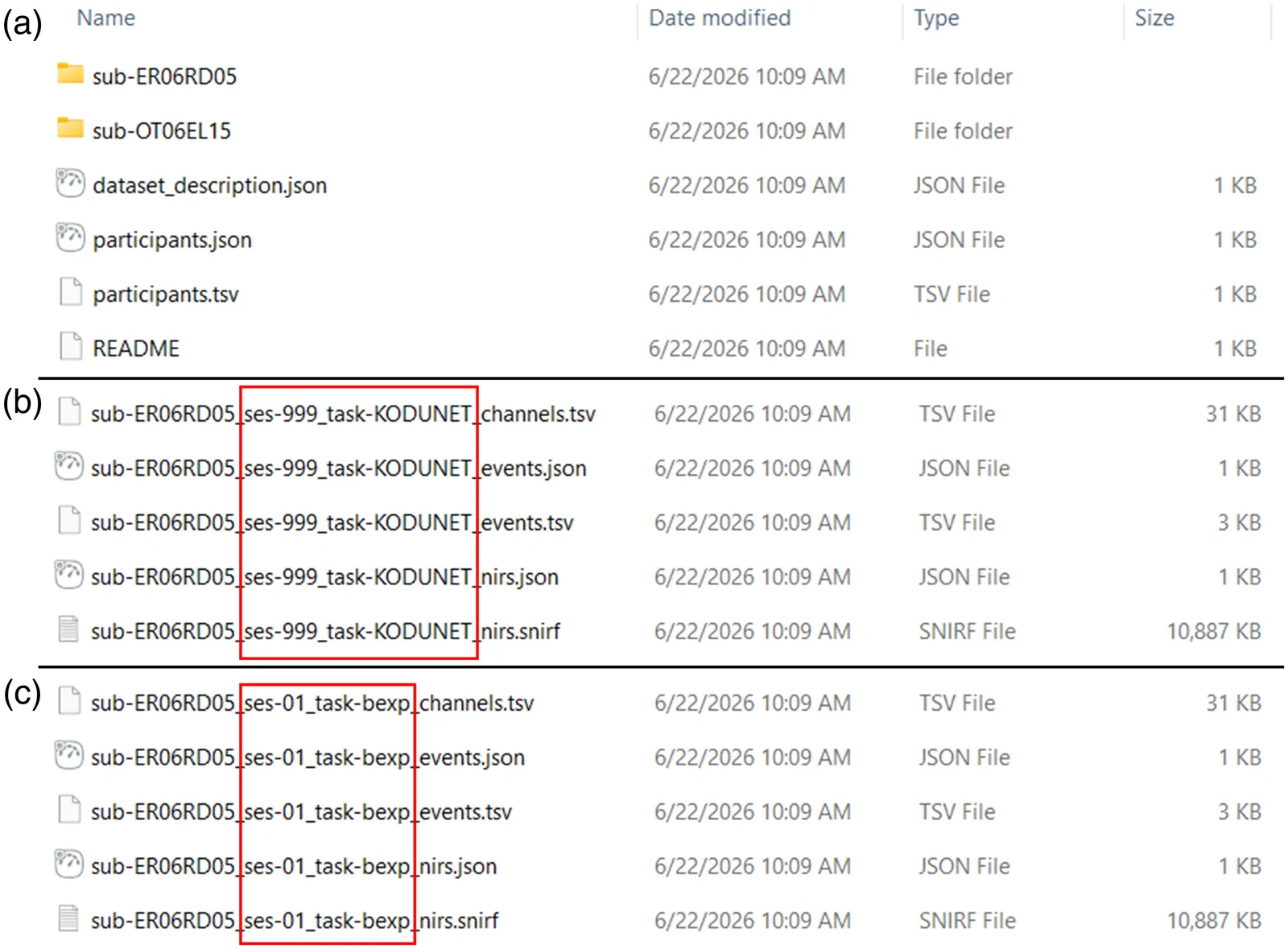
Overview of the no_mapping folder structure and renaming routine. (a) Top-level directory containing one subfolder for each participant. (b) Within each participant folder, the converted files are stored in the “ses-999/nirs” subdirectory. (c) After correcting the naming information, the files can be moved to the appropriate location in the output dataset.

Furthermore, the folder is structured in a BIDS-compatible way, which means that the IDs of subjects with at least one measurement that could not be assigned to a specific task and session are immediately visible in the first level of subfolders (as presented in Fig. 10).

Within “unmapped_recordings.csv,” or after opening all subsequent subfolders until the “nirs” subfolder appears, the user will see the BIDS-formatted filenames for every affected measurement. From “ses-999,” it can be inferred that the measurement could not be assigned to any session. From “task-KODUNET,” it can be inferred that “KODUNET” was entered as an experiment name during the measurement. In that case, it is probable that an underscore was forgotten, as “KODUN_ET” would correspond to the “bexp” task during session 01. The user can now manually rename these files to match the information from the study. In our case, “ses-999” is replaced by “ses-01” and “KODUNET” is replaced by “bexp.” Subsequently, the files need to be copied into the correct subfolder of the main output folder. In the present case, it would be “output > sub-ER06RD05 > ses-01 > nirs.”

**Fig. 11:**
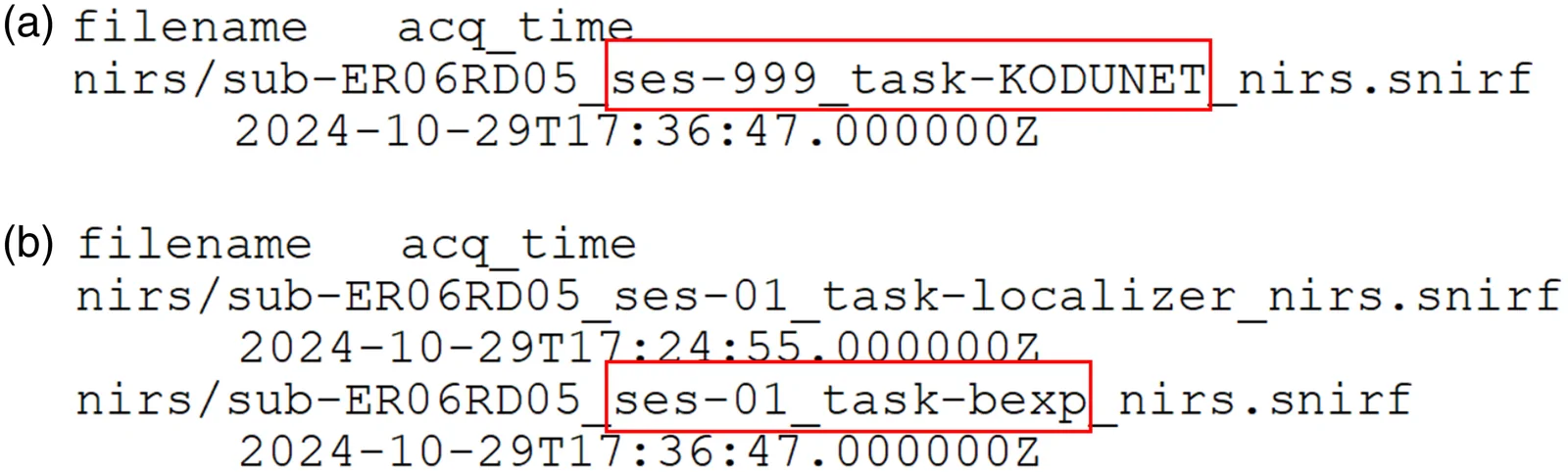
Manual correction of the session-specific *_scans.tsv file. (a) Entry corresponding to a recording stored in the no_mapping folder. (b) Corresponding entry in the successfully converted dataset that must be updated to include the unmapped recording.

Subsequently, the user also needs to open the *_scans.tsv file in the “ses-999” subfolder of each participant with a text editor and copy the corresponding entry to the “*_scans.tsv” file in the main output subfolder and change the session number and task name in the same way (see Fig. 11).

Once all naming conflicts have been resolved, it is important to manually delete the “no_mapping folder” as it is contained within the output folder, but should not be part of the final BIDS dataset.”

### 13.8 BIDS Validation

Once the dataset is converted, the user can now upload the output folder to the BIDS validator for confirmation of the BIDS structure.^34^ After having performed all previously conversion steps on the dataset, we are not getting any “error” message (color code red), which means that the dataset successfully complies with the BIDS standard.

**Fig. 12:**
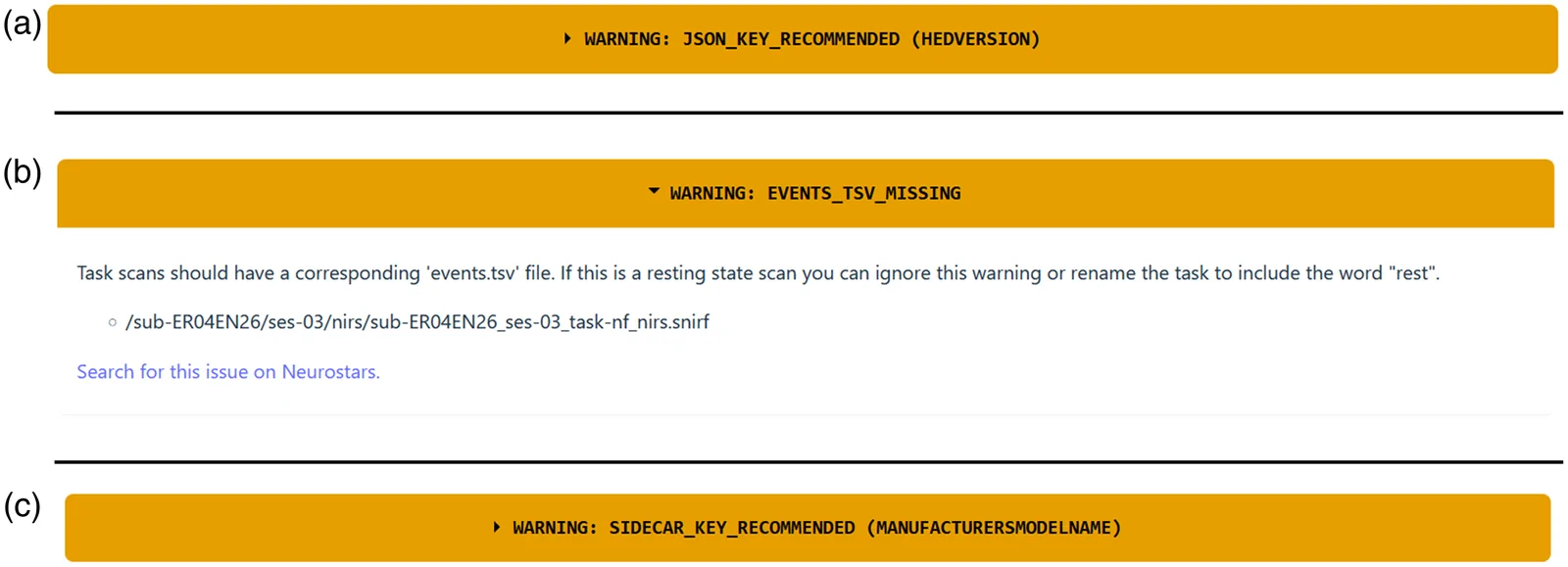
Example messages from the BIDS validator output. (a) Missing JSON keys. (b) Missing “*_events.tsv” file – BIDS validator text output is also shown. (c) Missing sidecar key.

However, some warnings appear (color code yellow), indicating that information recommended to be included in the BIDS nomenclature is not found. By clicking on the colored bar, the user sees which specific files are concerned by the warning message. This is expected as SNIRF2BIDS extracts all information required by BIDS, but only covers some of the recommended information. In our example, warnings are of one out of three types (see also Fig. 12 for a screenshot of typical BIDS validator warning messages).

• “JSON_KEY_RECOMMENDED,” which means that a specific entry from the “dataset_description.json” is missing. These are mostly top-level information about the dataset such as the version of the Hierarchical Event Descriptor (HED) tag or license type of the dataset. These tags that can be edited manually in the respective *.json-file.
• EVENTS_TSV_MISSING” indicates that, for task “nf” during session 03 for subject ER06RD05, no event-related information could be extracted by SNIRF2BIDS. This might be due to the fact that, during recording, event triggers were not recorded, for example, because of a technical problem. In most cases, such an error will severely limit the reusability of this specific recording. If applicable, this is relevant information to be recovered or added manually to the “Readme.MD” file, using any common text editor.
• “SIDECAR_KEY_RECOMMENDED,” hinting at recommended information missing in either the events sidecar file (“*_events.json”) or the NIRS sidecar file (“*_nirs.json”). These warnings might appear because some types of information are not automatically included in the original recordings by the manufacturer or by the user. During the internal conversion routine, SNIRF2BIDS can therefore not extract this information. The “*_events.json” file describes the content of the “*_events.tsv” file, which contains information about timing and properties of events such as stimuli presentation, participant responses or other time-dependent markers. The “*_nirs.json” file is NIRS-specific and has a mostly technical content, such as software version of the equipment that produced the measurements or device serial number.

## 14 Discussion and Conclusion

This paper presents a fully local R-based, GUI-driven approach for converting fNIRS data to the BIDS specification, which is adaptable to a wide range of experimental designs. The GUI-based workflow enables users to convert recorded datasets while simultaneously familiarizing themselves with the underlying BIDS principles and structure, thereby lowering the barrier to BIDS adoption.

In line with the rapid growth of fNIRS publications in recent years,^1^ SNIRF2BIDS can enable researchers to implement open data practices and share fNIRS datasets more easily, which will enhance the reproducibility of their studies and also facilitate large-scale and machine-learning based analyses. fNIRS is being increasingly adopted in cognitive neuroscience given its unique features compared with other modalities^2^—including its noninvasiveness, cost-effectiveness, and suitability for recordings outside of the laboratory.^2^^,^^11^^,^^34^^,^^35^ Although the introduction of the SNIRF format established a standardized framework for storing fNIRS recordings and metadata, the NIRS-BIDS specification further defines how such data can be organized within a unified and interoperable folder structure.^13^ However, the manual restructuring of SNIRF files into BIDS format remains tedious and error-prone, motivating the development of automated conversion tools.

Existing tools rely on cloud-based platforms, such as the BIDS fNIRS Cloud,^14^ or on script-based workflows implemented in Python toolboxes such as Cedalion^19^ and MNE-BIDS.^20^ These approaches are well-suited for researchers who prefer to restructure their data via Python code or who can upload the raw data to the cloud, if they prefer to use a GUI. With SNIRF2BIDS, we provide an offline tool targeted at researchers in the fields of psychology and neuroscience who wish to use a GUI-based offline tool, in the R environment, to standardize their fNIRS data. Using our tool does not require programming expertise or the upload of raw data to external servers, thereby improving accessibility, scalability, and data protection.

Although SNIRF2BIDS is not distributed as a standalone executable, its installation and deployment follow standard practices within the R ecosystem. For researchers already using R, operating the tool requires updating other packages before starting the setup process, installing SNIRF2BIDS and launching the graphical user interface from an R session.

Our tool automates major components of the BIDS conversion process, including file naming and metadata extraction. Nevertheless, user intervention is still required outside of the application for the creation of the input directory structure (depending on the manufacturer of the recording device) and for dataset validation. In its current version, SNIRF2BIDS does not yet provide a fully GUI-based end-to-end workflow.

SNIRF2BIDS focuses on automating steps that are technically more challenging (extracting NIRS metadata, formatting study-level information into JSON files) or prone to error in manual processing (renaming multiple files across subjects, sessions, and tasks can become exponentially more complex). In future versions of the tool, preparation of the input directory could be facilitated through a graphical user interface (GUI), which would semi-automatically guide users in arranging their files according to the required hierarchical structure (“folder” routine).

For recordings performed with the NIRSPort2 and similar devices that automatically generate a “_description.json” file, SNIRF2BIDS already provides a more fully automated workflow. We anticipate that, through feedback from the research community and open-source development, the workflow can be further adapted to reduce or eliminate the need for manual preparation of input data structures. Furthermore, vendors could adapt their recording procedures to better align with BIDS-compliant conversion in the future, such that the usability of SNIRF2BIDS may be further improved across a broader range of acquisition systems.

In the future, it would be also greatly beneficial if a wider range of naming patterns could be automatically recognized, separately provided, or interactively prompted. For example, if a user had a SNIRF dataset already following the BIDS naming conventions, but missing the hierarchical folder structure and accompanying .tsv or .json files, future versions of SNIRF2BIDS could generate the folder structure based on the BIDS compliant filenames and directly jump to the generation of metadata files without requiring a manual restructuring of the input data.

Interactive, manual editing of incomplete original metadata is also beyond the present scope of the tool but represents a potential direction for future extensions. Currently, SNIRF2BIDS shifts error handling back to the user, when specific files do not match the task mapping during conversion with the “json” routine. We describe the workflow for manually handling such cases in our end-to-end example. As recorded SNIRF files might miss required fields or have an invalid structure, SNIRF2BIDS currently displays error messages appearing during conversion to the user and saves them to a logfile in the output directory. These messages are informative, but interpreting them may require some background knowledge about SNIRF files and programming in R and Python. These additional steps during error handling currently represent a limitation for the usability of our tool.

In future versions, an interactive file viewer could streamline the handling of invalid files as well as detection of invalid IDs, enabling users to quickly review, edit metadata entries, or move these files directly within the app. Users could be guided through handling these files with more informative error messages. Furthermore, similar to existing R packages for organizing fMRI data that include interactive data quality inspection tools,^36^ future versions of SNIRF2BIDS could integrate visualization modules to facilitate fNIRS data quality assessment.^37^^,^^38^

Beyond technical considerations, ethical and legal challenges remain critical barriers to data sharing. Data sharing policies vary across countries and institutions, potentially imposing additional administrative and legal requirements, particularly regarding the geographical location of data repositories. Moreover, data from certain populations cannot always be shared, and the inclusion of secondary data—such as demographic variables, behavioral measures, multimodal recordings, or photogrammetry-based optode registration images—may substantially increase the risk of participant re-identification. Finally, transparent communication of data sharing policies prior to study participation remains essential to ensure informed consent. Notwithstanding these considerations, we encourage the conversion to BIDS-NIRS and data sharing using any pipeline to enhance reproducibility, dissemination of original data, and transparency and openness in our field.

Taken together, within the current framework of open science initiatives in the global fNIRS community, SNIRF2BIDS represents a user-friendly and privacy-conscious addition to existing toolchains, facilitating standardized data sharing and promoting reproducible research practices.

## Supplementary Material

10.1117/1.NPh.13.S3. S32603.s01

## Data Availability

All data and code reported in this paper are openly available on Github: https://github.com/raphael-lorenzdelaigue/snirf2bids.
