# Supplementary material for: SNIRF2BIDS: a GUI-based tool for converting functional near-infrared spectroscopy data to the Brain Imaging Data Structure in R
Source: Neurophotonics. 2026 Sep 17;13(Suppl 3):S32603. doi: 10.1117/1.NPh.13.S3.S32603 (PMC13585469; doi:10.1117/1.NPh.13.S3.S32603)

Code S1

Manual installation guide for R access to MNE-BIDS functions

```
Run this script in R to configure SNIRF2BIDS and its dependencies


# snirf2bids: Conversion of fNIRS to BIDS
#
# /by Lorenz-de Laigue, Svaldi, & Schroeder
#
# Manual configuration notes
#
# This R-script prepares your machine and configures a number of
dependencies
# step-by-step to use MNE-BIDS (Python) from R
#
# As a result, MNE-BIDS can be accessed from R and the snirf2bids
GUI can be used
#
# Alternatively, you can install snirf2bids from github after the
BiocManager and
# rhdf5 package had been configured
#
# Run this script only once; we install and configure:
# # Rhdf5 from Bioconductor to read and write *.snirf -files
# # An install of Miniconda (included in snirf2bids)
# # A new Conda environment that includes all packages that are
needed (included in snirf2bids)
#
# Dependent on your internet connection, this will take several
minutes

# (1) Get BiocManager and the rhdf5-package to read and write
*.snirf -files

if (!require("BiocManager", quietly = TRUE))
  install.packages("BiocManager")

if (!require("rhdf5", quietly = TRUE))
  BiocManager::install("rhdf5", configure.args="--no-staged-
install")

library(rhdf5)

# Now it is possible to install snirf2bids from github; to do so,
uncomment the following three lines:

#if (!require("pak", quietly = TRUE))
#  install.packages("pak")
#pak::pkg_install("raphael-lorenzdelaigue/snirf2bids")
```

```
# And then run this code to start the GUI
#library(SNIRF2BIDS)
#start_snirf2bids()

# The next steps can be skipped if using the auto-install from
snirf2bids

# (2) Get Reticulate to interface with Python and configure access
to MNE-BIDS

install.packages("reticulate")
library(reticulate)

# Install Miniconda into reticulate’s default location (no spaces in
path)
install_miniconda()

# Create a new conda environment with a specific Python version
## MNE BIDS Require python 3.10 or later
#
conda_create("mne-env", packages = c("python=3.10")) #future scripts
call to "mne-env"

# Install Python packages into that env via conda
conda_install("mne-env", packages = c("mne", "numpy", "scipy", "mne-
bids[full]", "matplotlib", "pandas"),
              channel = "conda-forge")


# (3) Basic example of usage

# connect previously created python environment + packages
use_condaenv("mne-env", required = TRUE)

# Now python modules are available in R
mne      <- import("mne")
np       <- import("numpy")
mne_bids <- import("mne_bids")

# select a *.snirf file for further processing
# load first file from list
raw = mne$io$read_raw_snirf(file.choose(), preload=F)
raw$info["line_freq"] = 50  # specify power line frequency as
required by BIDS (e.g., Germany has 50 Hz)

# Use MNE-BIDS function, e.g, plot sensors or show the optode
positions
raw$plot_sensors()
plt$show()
```

```
# for more script-based processing, consult "convert.R" in snirf2bids

# Thank you for choosing Open Science!
```

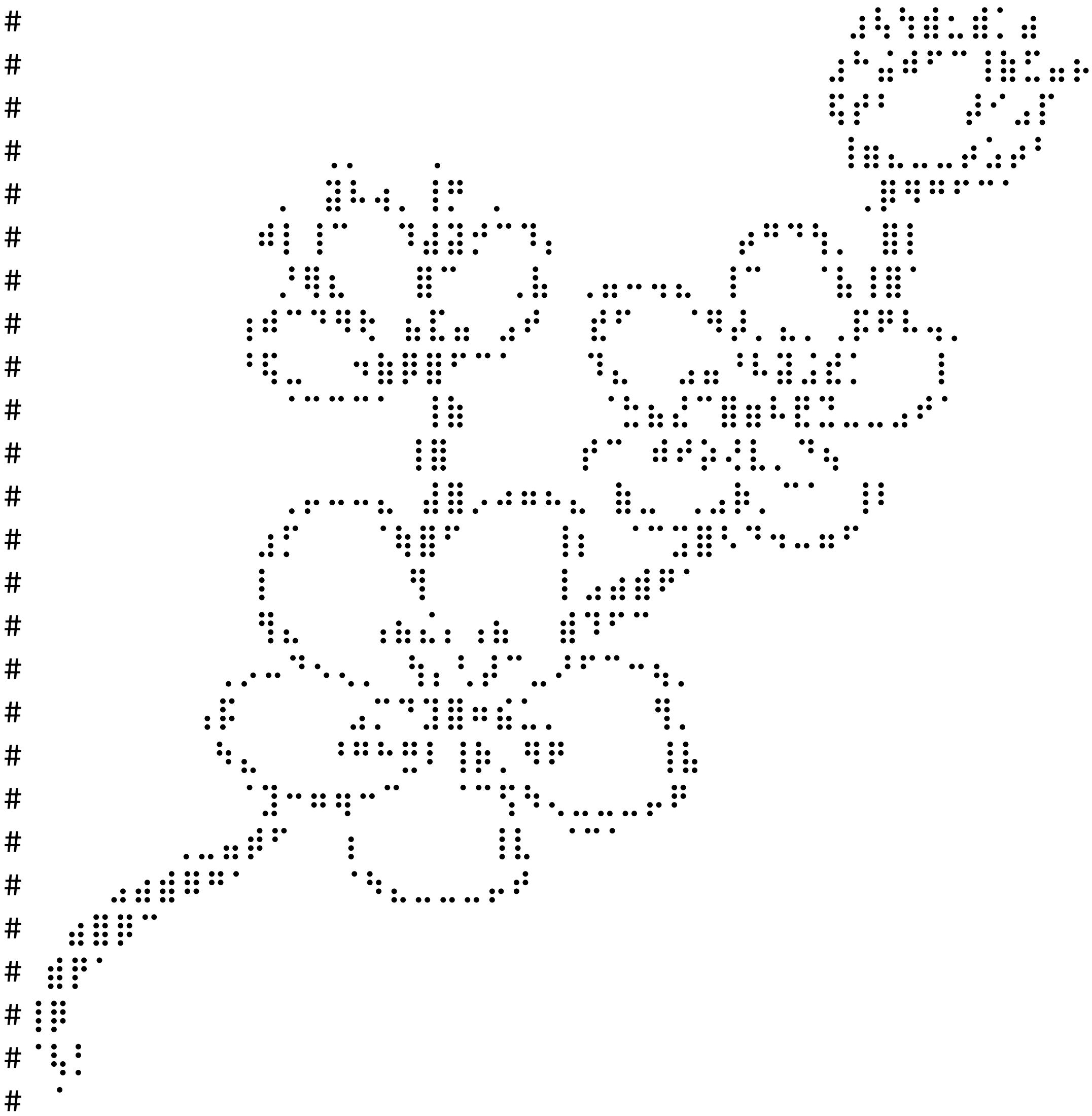

Supplement: Supplementary file 1 [file NPh_013_S32603_SD001.pdf]
